# Transcriptome-based analysis of human peripheral blood reveals regulators of immune response in different viral infections

**DOI:** 10.3389/fimmu.2023.1199482

**Published:** 2023-09-19

**Authors:** Sergey M. Ivanov, Olga A. Tarasova, Vladimir V. Poroikov

**Affiliations:** ^1^ Department of Bioinformatics, Institute of Biomedical Chemistry, Moscow, Russia; ^2^ Department of Bioinformatics, Pirogov Russian National Research Medical University, Moscow, Russia

**Keywords:** viral infections, transcriptomics, immune response, master regulators, peripheral blood mononuclear cells, COVID-19

## Abstract

**Introduction:**

There are difficulties in creating direct antiviral drugs for all viruses, including new, suddenly arising infections, such as COVID-19. Therefore, pathogenesis-directed therapy is often necessary to treat severe viral infections and comorbidities associated with them. Despite significant differences in the etiopathogenesis of viral diseases, in general, they are associated with significant dysfunction of the immune system. Study of common mechanisms of immune dysfunction caused by different viral infections can help develop novel therapeutic strategies to combat infections and associated comorbidities.

**Methods:**

To identify common mechanisms of immune functions disruption during infection by nine different viruses (cytomegalovirus, Ebstein-Barr virus, human T-cell leukemia virus type 1, Hepatitis B and C viruses, human immunodeficiency virus, Dengue virus, SARS-CoV, and SARS-CoV-2), we analyzed the corresponding transcription profiles from peripheral blood mononuclear cells (PBMC) using the originally developed pipeline that include transcriptome data collection, processing, normalization, analysis and search for master regulators of several viral infections. The ten datasets containing transcription data from patients infected by nine viruses and healthy people were obtained from Gene Expression Omnibus. The analysis of the data was performed by Genome Enhancer pipeline.

**Results:**

We revealed common pathways, cellular processes, and master regulators for studied viral infections. We found that all nine viral infections cause immune activation, exhaustion, cell proliferation disruption, and increased susceptibility to apoptosis. Using network analysis, we identified PBMC receptors, representing proteins at the top of signaling pathways that may be responsible for the observed transcriptional changes and maintain the current functional state of cells.

**Discussion:**

The identified relationships between some of them and virus-induced alteration of immune functions are new and have not been found earlier, e.g., receptors for autocrine motility factor, insulin, prolactin, angiotensin II, and immunoglobulin epsilon. Modulation of the identified receptors can be investigated as one of therapeutic strategies for the treatment of severe viral infections.

## 1 Introduction

Viruses cannot replicate without host cells; therefore, their interaction with hosts is crucial for viral replication, and transmission. On the other hand, the immune system defends the host organism from the virus. Thus, the development of viral infection and the severity of the disease is mediated by a complex interplay between the virus and host immune system (1–4). The intensity and efficacy of the immune response depends on the immune status of a person and the ability of viruses to disrupt human immunity (5). Therefore, the peculiarities of interactions between a particular virus and the human body and corresponding individual host response to infection can be substantial parts of a whole pathogenesis process that determine the severity of viral diseases.

Several viruses, e.g., cytomegalovirus (CMV), Epstein-Barr virus (EBV), human T-cell leukemia virus type 1 (HTLV-1), hepatitis B and C viruses (HBV and HCV), human immunodeficiency virus 1 (HIV-1), can cause chronic or latent infections. Herpesviruses, including CMV and EBV, cause latent infection and can persist for a long time in healthy people without clinical symptoms, but they can be reactivated by inflammatory stimuli or immunosuppression and cause various pathologies (6, 7). Retroviruses such as HTLV-1 and HIV-1 cause chronic infections by integrating viral genome into human DNA. HTLV-1 usually persists in a human organism without symptoms; however, it may cause adult T cell leukemia/lymphoma or HTLV-1-associated myelopathy/tropical spastic paraparesis in a small part of infected people (8, 9). HIV-1 may also persist in the human organism without symptoms for many years but eventually causes acquired immunodeficiency syndrome in almost all people living with HIV and do not receiving antiviral treatment (10).

Other viruses from various families, such as coronaviruses, influenza, and Dengue virus (DENV), cause acute infection diseases with different degrees of severity. In this case, the disease may lead to death or recovery with the complete elimination of the virus (11, 12).

Different viruses, including new, suddenly arising ones, such as SARS-CoV-2, have different transmission routes and target cells, and causes infectious diseases of different duration and severity; therefore, it is challenging to create direct antiviral therapeutics due to differences in the structures and functions of viral targets and the long duration of the drug development process. Although drugs with specific direct antiviral activity (13) including inhibitors of viral nucleic acid polymerases, proteases, integrases, etc., are available for hepatitis C virus (14, 15) and HIV (16), their use may be limited by drug resistance, side effects and toxicity, especially in pediatric and geriatric patients (17), pregnant women (18, 19) and people after surgery. Accessibility of medicines (15, 20) and patient adherence (21) are important determinants of treatment efficacy. Antiretroviral drugs, even if they lead to an undetectable viral load, do not provide the elimination of the virus. Latent HIV infection is another problem that can reduce the effectiveness of drug treatment (10). Thus, pathogenesis-directed therapy can be beneficial for treatment of severe viral infections and associated comorbidities. Despite significant distinctions in the pathogenesis of viral infections, many of them induce the dysfunction of the immune system (22–32). Long-lasting activated immune response with release of cytokines in the case of chronic infections or cytokine storm in the case of some acute infections, e.g., dengue fever (DF) and Coronavirus disease 2019 (COVID-19), lead to cytokine-dependent immune activation, which, in turn, causes immune dysfunction, the so-called “exhaustion”, and significant loss of T lymphocytes. As a result, the immune system cannot eliminate viruses effectively, which leads to high severity of disease, duration, and development of secondary infections and other comorbidities. For instance, chronic cytokine-dependent activation of T cells by HIV-infection causes their apoptosis followed by dysfunction of immune response to infection (33). CMV increases disease progression and mortality in infants with HIV-infection since it raises activation and apoptosis of both CD4(+) and CD8(+) T cells (16). COVID-19 and dengue hemorrhagic fever (DHF) are associated with cytokine storm, which causes the induction of T lymphocytes apoptosis, increasing the disease severity (31, 33, 34). Thus, it is crucial to identify common pathogenetic mechanisms for different viral infections because it will allow developing of new anti-viral drugs applicable for the treatment of severe viral infections.

One of the efficient ways to identify common mechanisms of human immune functions alteration in response to a viral infection is an analysis of genome-wide data obtained by high-throughput experimental methods (10). Most of the studies that analyzed virus-host interactions use the transcriptomics data obtained by microarray and RNA sequencing approaches (35–48). Some of these studies focused on comparing transcriptional changes in blood cells caused by the same or similar viral infections, e.g., respiratory viral infections. For instance, Vavougios G.D. analyzed transcriptomics data and found overlapping host gene signatures between SARS-CoV-2 and other viral and bacterial potential copathogens (40). Dunmire S.K. with colleagues compared the EBV transcription response profile to multiple other acute viral infections, including influenza A, respiratory syncytial virus, human rhinovirus, attenuated yellow fever virus, and DENV. They revealed similarity only to DENV profile as well as that of presented in patients with hemophagocytic syndromes, suggesting that EBV and DENV cause uncontrolled inflammatory responses (35). McClain M.T. with colleagues measured transcription profiles from COVID-19 patients and directly compared them to subjects with seasonal coronavirus, influenza, bacterial pneumonia, and healthy controls. They identified a 23-gene signature, which can be utilized to diagnose and differentiate COVID-19 from other viral diseases (43). Tsalik E.L. and colleagues analyzed gene expression in blood cells and identified transcriptional changes that are either virus-specific or common to respiratory infections such as influenza, enterovirus/rinovirus and human metapneumovirus infections, and nonrespiratory DENV infection (46). Avraham Unterman and co-authors used gene expression analysis of peripheral blood mononuclear cells (PBMC) and searched for protein biomarkers of COVID-19 progression. As a result, the significant increase of interferon-I response and amphiregulin expression levels was found in patients with COVID-19 comparing to the healthy controls. Moreover, some regulatory and tissue repair-associated genes (CD163, IL1R2, AREG, HAVCR2 (encoding TIM-3), and its ligand LGALS9) and pathways (TIM-3/Gal-9 pathway) were found up-regulated in progressive COVID-19 patients (47). Zhidong Tang and co-authors developed the portal of viral infection containing data on dynamic changes and differential expression profiles of genes in viral-infected patients (48).

The current study aimed at the analysis of transcriptomics data for viral diseases focusing on causes for alteration of immune functions. We used the following two criteria for selecting viruses for the analysis. First, we chose the viruses based on their significance to human health, particularly the harm they can cause due to immune dysfunction. Second, we chose the viruses for which there are publicly available transcription level data of blood cells obtained from both viral-infected and uninfected people who did not take any antivirals. Consequently, we obtained transcriptomic data sets of nine viruses. We compared the transcription profiles in blood cells between pathologies caused by nine viruses (49–55). They all cause immune dysfunction, and transcription changes in blood cells may reflect alterations in their functions. Since human peripheral blood cells are typically involved in the host response to a particular viral infection, we suppose that the observed changes in the level of transcription of these cells can represent the most significant peculiarities of immune response to the infection. The corresponding transcriptomics data, measured under the same conditions, including cell types, are available in public databases. We used originally developed pipeline for analysis of transcriptome data collected from patients with several viral infections to reach deep understanding of molecular mechanisms leading to immune dysfunction that can be common for the analyzed infections. The pipeline includes the following steps: (1) collection of transcriptome data; (2) data processing and normalization; (3) data analysis and search for master regulators that can be responsible for altered immune response. In contrast to earlier published studies, we identified not only common differentially expressed genes (DEGs), variations in the interplay of pathways, but also master regulators (MRs), which are the proteins at the top of signaling pathways responsible for the observed transcription changes. Particularly, we found receptors for cytokines, growth factors, hormones, and mediators, which are potentially important regulators for altered immune functions in most of viral diseases caused by nine viruses.

## 2 Materials and methods

### 2.1 Collection of transcription datasets

We performed a rigorous search across two transcriptomics databases: Gene Expression Omnibus^1^ (GEO), and ArrayExpress^2^ using the following query: “(virus OR viral) AND (lymphocytes OR “B cells” OR “T cells” OR monocytes OR “NK cells” OR PBMC OR “peripheral blood mononuclear” OR neutrophils OR “whole blood”).” We selected datasets containing data on gene transcription in blood cells measured by microarrays or RNA sequencing and obtained from both viral-infected and uninfected people. We selected only datasets obtained from people who did not take antiviral therapeutics. In total, we identified 112 data sets, which were different by the virus, cell type, and experimental method. The most frequently used cell type was PBMC. The datasets related to these cells contain transcription profiles from patients infected by SARS-CoV-1, SARS-CoV-2, DENV, HIV-1, HTLV-1, HBV, HCV, CMV, and EBV.

### 2.2 Genome enhancer pipeline

#### 2.2.1 Main steps of the analysis

To identify DEGs and MRs, we used the Genome Enhancer tool^3^ developed by geneXplain GmbH (56). Briefly, Genome Enhancer implemented a pipeline including four main steps:

1. Pre-processing of gene transcription data and identification of DEGs.
2. Identification of the regulatory regions (promoters and enhancers) of the DEGs;
3. Analysis of the regulatory regions of DEGs to predict transcription factor binding sites (TFBSs), identify those enriched in promoters and enhancers of DEGs compared to genes with unchanged transcription, and predict the combinations of TFBSs called “composite regulatory modules” (CRM), which may reflect complexes of co-acting TFs;
4. Reconstruction of the signaling network that activates these TFs and identify MRs at the top of the network.

#### 2.2.2 Pre-processing of gene transcription data and identification of differentially expressed genes

The initial files downloaded from GEO were transformed to tables using functions from Affy and limma R packages, including ReadAffy, read.idat and read.maimages for Affymetrix, Illumina and Agilent platforms, correspondingly. All RNA sequencing datasets were presented as read counts tables and did not require transformation. All microarray datasets were normalized using rma, neqc, and normalizeBetweenArrays functions, for Affymetrix, Illumina and Agilent platforms, correspondingly. Obtained tables were loaded into Genome Enhancer pipeline.

The first stages of Genome Enhancer pipeline are related to additional data processing. Since subsequent stages of the pipeline and comparison of viral-induced transcription profiles between different microarray platforms require data at the level of genes instead probes, several probe intensity values were merged into single value. If the gene was associated with two or more probe intensity values, the maximal value was chosen. As a result, microarray probe ids were converted to ensemble gene ids. All table were additionally normalized using quantile normalization method.

The DEGs were identified between viral disease samples and healthy controls for each of the nine viruses separately. Genome Enhancer allowed identifying DEGs using limma R package for microarray data and edgeR package for RNA sequencing data. For each dataset, the top 300 up-regulated and top 300 down-regulated genes were used for subsequent steps of Genome Enhancer pipeline (see Sections 2.2.3, 2.2.4, 2.2.5).

To perform pathway enrichment analysis and compare transcription profiles between viral infections at the level of particular genes, we defined up- and down-regulated genes with absolute values of log fold changes (log2 values) exceeded 0.5 and p-values less than 0.05. We did not use Benjamini–Hochberg or Bonferroni correction for multiple hypothesis testing because no DEGs for DENV, HTLV-1 and HIV-1 infections were found with any thresholds on adjusted p-values. Moreover, different microarray platforms allow measuring transcription of different numbers of genes, and corresponding correction will be different for each transcriptional dataset that may introduce additional bias into results of analysis.

#### 2.2.3 Identification of the regulatory regions of DEGs

The regulatory regions of DEGs were considered as promoter sequences of length of 1100 base pairs (bp): from 1000 bp before transcription start site (TSS) to 100 bp after TSS

#### 2.2.4 Identification of transcription factors and composite regulatory modules

The identified regulatory regions were then scanned for TFBS and their combinations – CRM, which were enriched in promoters of top 300 up- and top 300 down-regulated genes compared to genes whose transcription was not changed. The TFBS were predicted using positional weight matrices from the TRANSFAC database (57), the CRM were identified using Composite Module Analyst algorithm (58). The corresponding analysis was performed for up- and down-regulated genes separately.

#### 2.2.5 Identification of master regulators

To identify MRs in signaling network, upstream of the identified transcription factors, the comprehensive network from TRANSPATH database (59) and “Upstream Analysis” algorithm (60, 61) were used. TRANSPATH database contains directed interactions between proteins, e.g., the directed interaction between two kinases where the first kinase phosphorylates the second kinase. “Upstream Analysis” algorithm scores potential MRs according to their closeness in network to transcription factors. As a result, we identified two lists of potential MRs: for up- and down-regulated genes, correspondingly.

To identify receptors among revealed MRs, we retrieved a list of receptors from the OmniPath database (62) using the OmnipathR R package.

### 2.3 Pathway enrichment analysis

To identify KEGG pathways^4^ that were differentially regulated in PBMC from a particular viral infection-related group of patients compared to healthy control, we performed pathway enrichment analysis (63) using the “enrichr” function from the “enrichR” R package (64). Function “enrichr” allows identifying pathways “enriched” with DEGs compared to the random gene sets. It means that the percent of genes from studied gene list participating in particular pathway is statistically significantly higher than the percent of genes from a random gene set. We performed the corresponding analysis for up- and down-regulated genes separately. For further analysis we selected pathways associated with at least three DEGs and with an adjusted p-value, calculated by Benjamini–Hochberg method, less than 0.05.

### 2.4 Comparison of KEGG pathways, functionally related groups of genes, and master regulators between viral infection diseases

To compare lists of KEGG pathways, manually selected groups of genes, and MRs obtained for infectious diseases caused by nine viruses, we created heatmaps using the “pheatmap” R package^5^.

## 3 Results

### 3.1 Data on viral-related gene transcription in peripheral blood mononuclear cells

We performed a comprehensive search across transcriptomics databases and selected 10 transcription datasets from the Gene Expression Omnibus (GEO) database containing data on gene transcription in PBMC from healthy people and patients infected by one of the nine viruses (see **Table 1**). The details are provided in the Materials and Methods section. Some datasets contain samples from patients with a different form of viral infection disease, e.g., different severity (DF or DHF, severe or moderate COVID-19), or duration of disease (acute or latent EBV and CMV infections). Further analysis was performed separately for each of these viral infection-related groups. We defined “viral infection-related group” as a group of samples obtained from a specific transcription dataset and related to the viral infection in a particular form (e.g., severe or moderate, acute or chronic, symptomatic or asymptomatic).

**Table 1:** Transcription datasets with information on gene transcription in PBMC derived from healthy people and patients infected by one of 9 viruses.

Virus	Dataset	Patient groups	Healthy controls	Platform	Reference
Cytomegalovirus (CMV)	GSE81246	Samples from CMV seropositive (n=25) healthy donors and people with symptomatic primary CMV infection (n=11)	Samples from CMV seronegative healthy donors (n=12)	Affymetrix HuGene-2_1-st	(65)
Dengue virus (DENV)	GSE18090	Samples from patients with DHF (n=10) and classical DF (n=8)	Samples (n=8) from febrile patients confirmed to be not infected with DENV	Affymetrix HG-U133 Plus_2	(52)
Epstein–Barr virus (EBV)	GSE45919	Samples from people during acute EBV infection (n=3) and during latency (n=12)	Samples from people before acquisition of EBV (n=9)	Illumina HumanHT-12 V4.0	(35)
Hepatitis B (HBV)	GSE58208	Samples from chronic HBV carriers (n=12)	Healthy people (n=5)	Affymetrix HG-U133 Plus_2	–
Hepatitis C (HCV)	GSE40184	Samples from patients infected with HCV (n=10)	Healthy people (n=6)	Affymetrix HG-U133A	(50)
Human immunodeficiency virus (HIV)	GSE77939	Samples from untreated people living with HIV (n=5)	Healthy people (n=4)	Affymetrix PrimeView	(54)
Human T-cell leukemia virus type 1 (HTLV-1)	GSE55851	Samples from HTLV-1 carriers (n=6)	Healthy people (n=3)	Agilent 026652 4x44K v2	(51)
SARS-CoV-1	GSE1739	Samples from patients with SARS (n=10)	Healthy people (n=4)	Affymetrix HG-Focus	(53)
SARS-CoV-2	GSE152418	Patients with COVID-19: patients in Intensive Care Unit (n=4), patients with severe disease, but not in Intensive Care Unit (n=8), patients with moderate disease (n=4)	Healthy people (n=17)	Illumina NovaSeq 6000	(49)
SARS-CoV-2	GSE179627	Samples from patients with COVID-19 (n=13)	Healthy people (n=22)	Illumina NovaSeq 6000	(55)

Up-regulated genes for each viral infection-related group (**Table 2**) were defined as having log fold changes more than 0.5 and p-values less than 0.05. Down-regulated genes for each viral infection-related group were defined as having log fold changes less than -0.5 and p-values less than 0.05. These thresholds were selected empirically to balance the numbers of DEGs and their statistical significance (see section 2.2.2). In total, 16129 DEGs were identified. The full lists of DEGs presented in **Tables S1**, **S2**.

**Table 2:** Numbers of up- and down-regulated genes associated with viral infection-related groups.

Sample group	Abbreviation	Samples	Controls	Up- regulated^1^	Down-regulated^1^	Interferon-stimulated genes ^2^
Cytomegalovirus primary infection	CMV_pr	11	12	833 (792)	943 (888)	10 (p <10^-4^)
Cytomegalovirus seropositive healthy donors	CMV_pos	25	12	8 (0)	8 (0)	0
Ebstein-Barr acute infection	EBV_a	3	9	2343 (436)	2373 (291)	20 (p <10^-4^)
Ebstein-Barr latent infection	EBV_l	12	9	3255 (3166)	3315 (3201)	11 (p = 0.4)
Hepatitis B chronic infection	HBV	12	5	936 (666)	1097 (894)	12 (p <10^-4^)
Hepatitis C chronic infection	HCV	10	6	136 (89)	308 (216)	7 (p <10^-4^)
Human T-cell leukemia virus type 1 infection	HTLV_1	6	3	430 (0)	495 (0)	2 (p = 0.12)
Human immunodeficiency virus infection	HIV	5	4	192 (0)	108 (0)	18 (p <10^-4^)
Dengue hemorrhagic fever	DHF	10	8	878 (7)	608 (5)	14 (p <10^-4^)
Dengue fever	DF	8	8	384 (0)	368 (0)	24 (p <10^-4^)
Severe acute respiratory syndrome	SARS	10	4	720 (79)	836 (95)	3 (p = 0.88)
Coronovirus Disease 2019 (COVID-19) - patients with evident clinical symptoms	COVID19	13	22	1605 (1554)	2004 (1925)	29 (p <10^-4^)
Coronovirus Disease 2019 (COVID-19) - patients in Intensive Care Unit (ICU)	COVID19_ICU	4	17	2925 (2751)	2640 (2204)	7 (p = 0.76)
Coronovirus Disease 2019 (COVID-19) - patients with severe disease, but not in Intensive Care Unit (ICU)	COVID19_Sev	8	17	2104 (1899)	1698 (1331)	18 (p <10^-4^)
Coronovirus Disease 2019 (COVID-19) - patients with moderate disease	COVID19_Mod	4	17	1699 (1438)	1562 (1108)	31 (p <10^-4^)

^1^Numbers of up- and down-regulated genes with |logFC| > 0.5 and unadjusted p-value < 0.05. The corresponding numbers of genes with adjusted p value < 0.05 are shown in brackets.

^2^Numbers of up-regulated genes intersected with the list of 50 interferon-stimulated genes along with statistical significance of intersections (p values).

**Figure 1:**
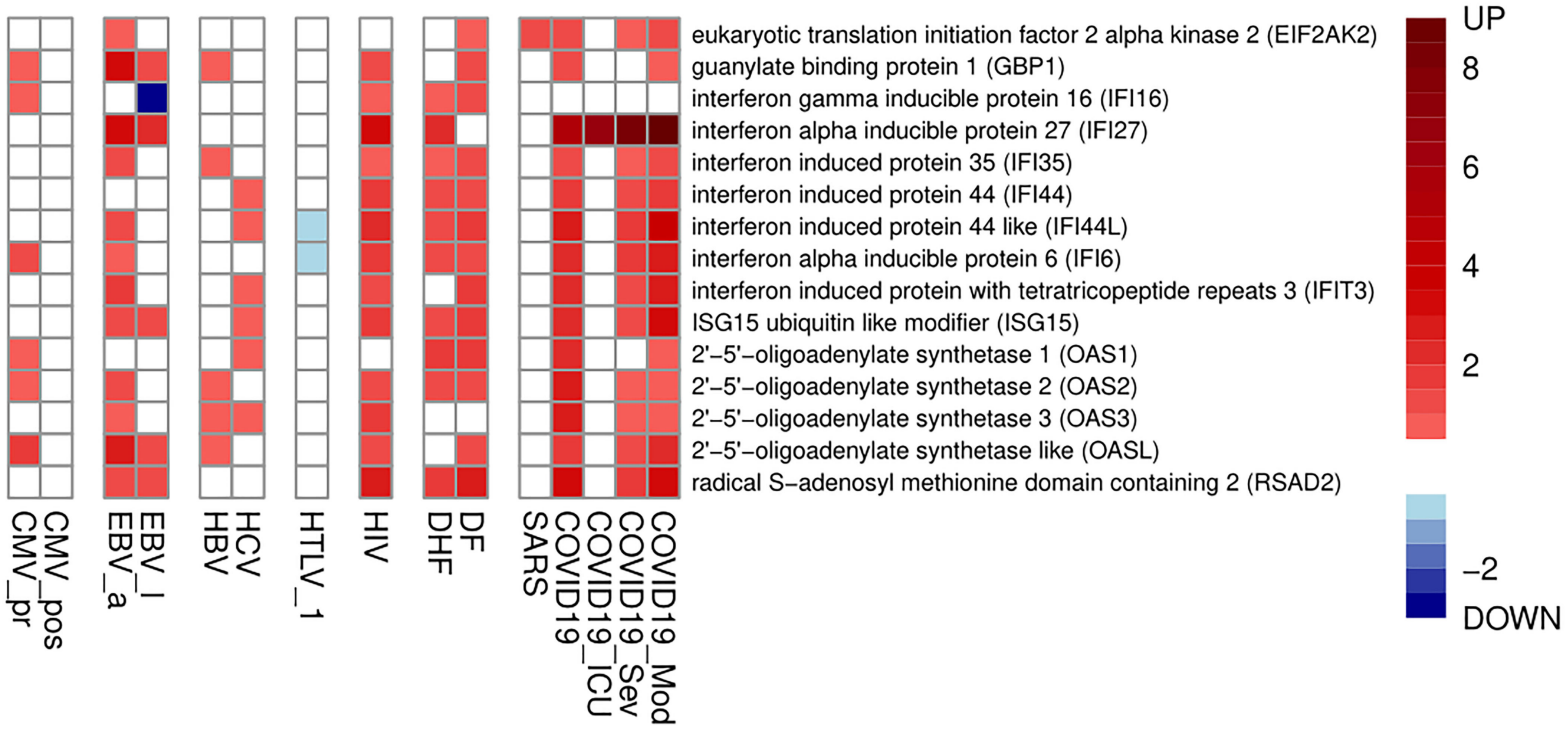
Interferon-stimulated genes, which are up- or down-regulated in at least four viral infections. The color legend shows logarithm of fold change of gene expression.

Since most of viruses trigger the type I interferon system, we compared the obtained lists of up-regulated genes with the list of 50 interferon-stimulated genes (ISGs) from review of John Schoggins and Charles Rice (66) as a control (see **Table S3**). We calculated Tanimoto coefficients to estimate the gene lists intersections and performed permutation tests to estimate their statistical significance. The intersections of up-regulated genes with known ISGs were significantly high for the most of viral infection-related groups except CMV_pos, HTLV_1, SARS and COVID19_ICU (**Table 2**) (see Discussion for details). **Figure 1** shows the ISGs that were up- or down-regulated in at least four viral infections.

We considered a gene up- or down-regulated by a particular viral infection if it was up- or down-regulated in at least one of viral infection-related groups, e.g., DF or DHF. We found no genes that were up- or down-regulated by all nine or eight viral infections, and there were only four genes (B4GALT5, CEBPD, H2AC8, PLAUR) differentially regulated in seven viral infections, whereas direction of changes of their expression (up- or down-regulation) depended from particular infection (see **Table S2**). The observed small intersections between DEGs may be explained by differences in the pathogenesis of viral infections and differences in experimental methods, microarray platforms, and batch effects. We suggested analysing the similarity of viral infections at the level of both individual genes and biological pathways.

### 3.2 Identification for individual genes and biological pathways that are common for different viral infections

**Figure 2:**
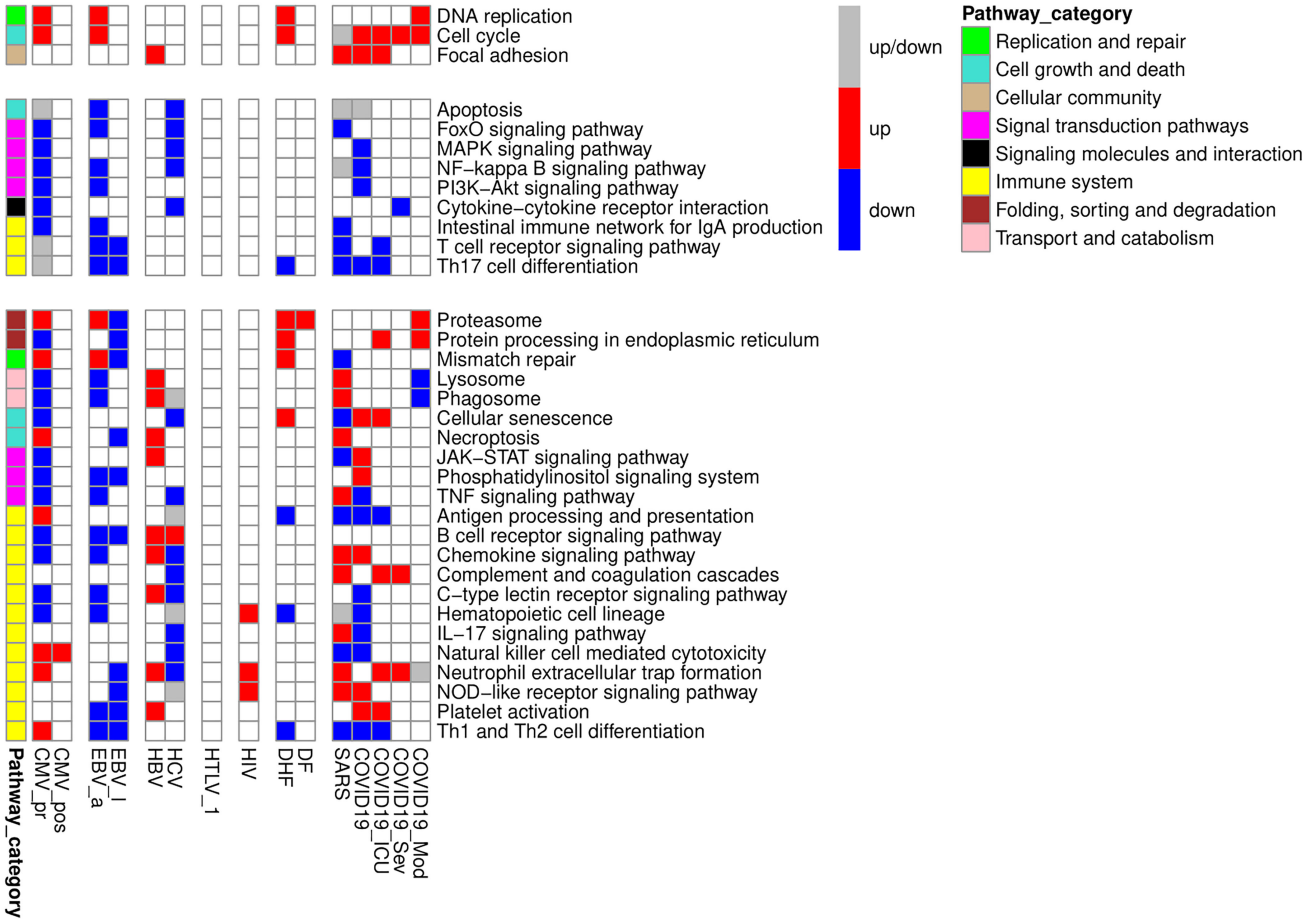
KEGG pathways, up- or down-regulated in PBMC by at least three viral infections. The figure is divided into three parts containing genes which are only up- or down-regulated by viral infections, and genes with mixed pattern of expression changes. The red color of the cell means that the pathway is enriched by up-regulated genes, the blue color of the cell means that the pathway is enriched by down-regulated genes, grey color of the cell means that the pathway is enriched by both up- and down-regulated genes. Pathway category is a related group of pathways according to pathway classification from KEGG PATHWAY database.

We performed a pathway enrichment analysis (see Materials and Methods) to identify KEGG pathways, which are up- or down-regulated in PBMC during studied viral infections. We considered a pathway to be “enriched” with up- or down-regulated genes, if at least three genes from studied gene list were in the pathway and corresponding proportion of genes was statistically significantly higher than the proportion from a random gene set (adjusted p-value less than 0.05). A pathway was considered as differentially regulated by a particular viral infection if it was differentially regulated in at least one viral infection-related group regardless of the direction of regulation (up- or down-regulation). We created a heatmap (**Figure 2**) demonstrating pathways, which were differentially regulated by at least three of nine viral infections. The pathways in the **Figure 2** were grouped by categories from KEGG PATHWAY database. The full list of pathways is presented in **Table S4**.

We manually investigated the positions of DEGs in the pathway maps and selected genes, which characterize the functional states of PBMC in studied viral infections. Since not all DEGs were presented in KEGG pathways, we also manually investigated functions of genes, which were up- or down-regulated in five or more viral infections, using information from Uniprot database^6^. We found that most of up-regulated genes belong to two large groups: genes related to cell division and immune-specific functions. Genes related to cell division include key regulators of cell cycle (e.g., cyclins and cyclin-dependent kinases), checkpoint kinases, centrosome, cytoskeletal and motor proteins, enzyme required for DNA replication and repair. The down-regulated genes include immune-related genes and genes related to the processing of genetic information: transcription factors, regulators of splicing, RNA modification and translation.

**Figure 3:**
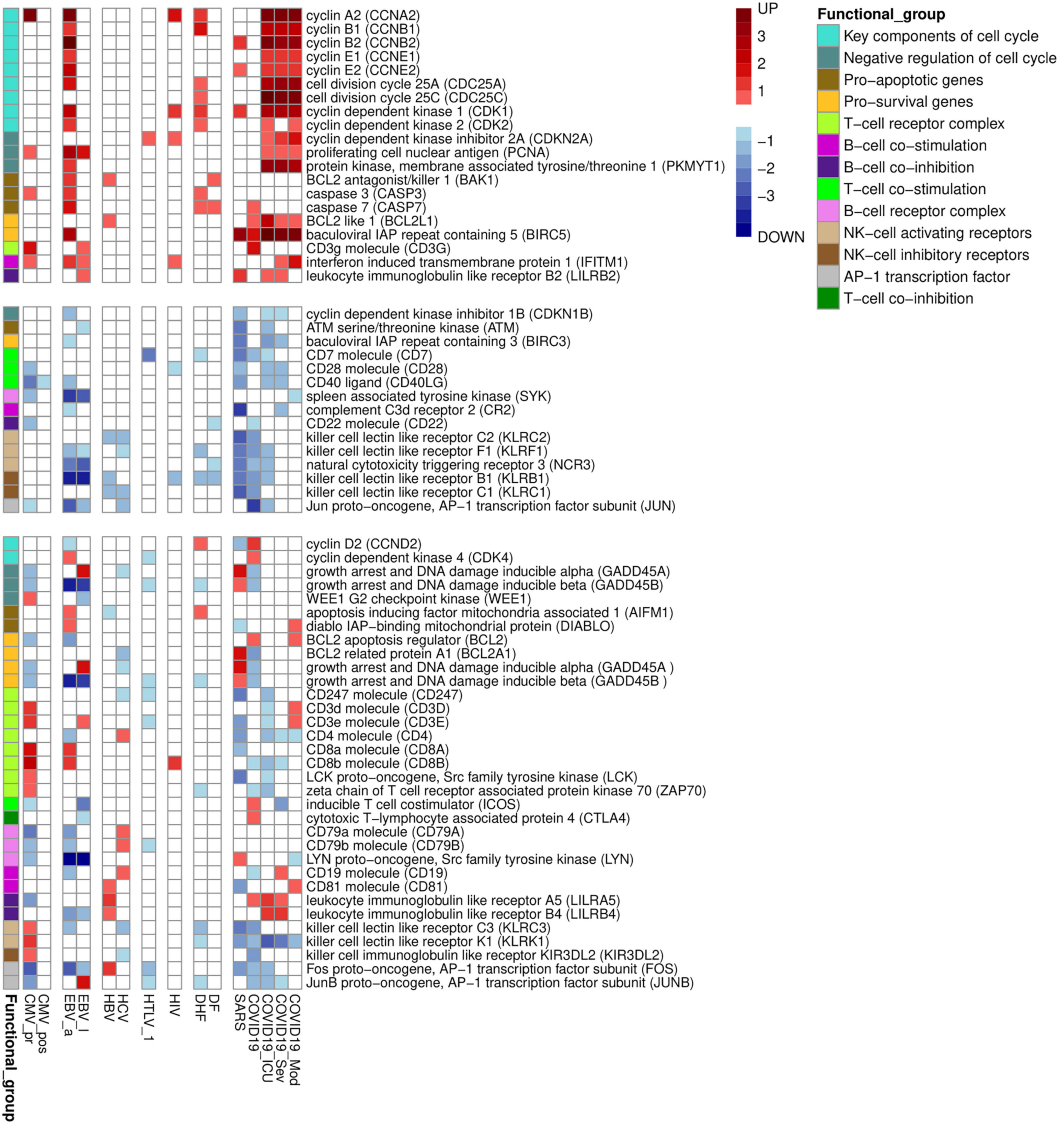
Functionally related groups of genes, up- or down-regulated in PBMC by at least three viral infections. The figure is divided into three parts containing genes which are only up- or down-regulated by viral infections, and genes with mixed pattern of expression changes. The color legend shows logarithm of fold change of gene expression. The functional group is a group of genes related to the particular cell process in PBMC.

We manually divided the DEGs into smaller functional groups reflecting functional state of immune cells (**Figure 3**). Along with key regulators of cell cycle and apoptosis, **Figure 3** contains key genes, which are well known positive and negative regulators of B, T, and NK cells activation. **Table 3** contains data on additional immune-related genes, whose relationships to viral infections are not as well described, and which are up- or down-regulated by at least five viral infections.

**Table 3:** Immune-related genes, which are up- or down-regulated in PBMC by at least five viral infections.

Gene	Immune function ^1^	CMVpr *	CMVpos	EBV_a	EBV_l	HBV	HCV	HTLV_1	HIV	Dengue_DHF	Dengue_DF	SARS	COVID19	COVID19_ICU	COVID19_Sev	COVID19_Mod
Up-regulated genes																
CD38	NAD+ glycohydrolase, a marker for immune cell activation	↑*		↑*	↓*				↑	↑	↑		↑*	↑*	↑*	↑*
CXCL10	Proinflammatory cytokine, involved in processes of chemotaxis, differentiation, and activation of immune cells	↑*		↑*			↑			↑	↑		↑*	↓*		↑*
LY6E	Surface protein that regulates T lymphocytes proliferation, differentiation and activation				↓*	↑					↑		↑*		↑*	↑*
SEC14L1	Protein that inhibits the antiviral RIG-I signaling pathway					↑*		↑		↑	↑	↑		↑*		
SECTM1	A ligand for CD7, costimulatory receptor for T cell proliferation, expressed by neutrophils and monocytes	↓*		↑	↑*	↑*	↑*								↑*	
SIGLEC1	Macrophage-restricted adhesion molecule that mediates sialic-acid dependent binding to various immune cells				↑*		↑*		↑		↑		↑*		↑*	↑*
Down-regulated genes																
CCR7	Receptor that activates B and T lymphocytes, controls the migration of memory T cells to inflamed tissues, stimulates dendritic cell maturation	↓*		↓					↓	↓	↓	↓	↑*	↓*	↓*	
CD83	Receptor that involved in the regulation of antigen presentation	↓*		↓						↓	↓		↓*	↓	↓*	
CD96	Potentially plays a role in the adhesive interactions of activated T and NK cells during the late phase of the immune response	↑*				↓				↓	↓	↓		↓*		
CXCR5	Chemokine receptor that involved in B-cell migration into B cell follicles of spleen and Peyer patches			↓*				↓			↓	↓*		↓*	↓	
FCMR	Fc receptor for IgM that protects immune cells from apoptosis			↓*					↓		↓	↓		↓*		
GPR183	Chemotactic receptor for B cells, T cells, splenic dendritic cells, monocytes/macrophages and astrocytes	↓*		↓*						↓	↓	↓		↓	↓*	
IL13RA1	The subunit of receptor for interleukin 13 that down-regulates macrophage activity, inhibits the production of pro-inflammatory cytokines and chemokines, and promotes IgE isotype switching of B cells	↓*		↓	↓*					↓		↑	↓*			↓*
IL1B	Pro-inflammatory cytokine that is the major endogenous pyrogen. Induces prostaglandin synthesis, neutrophil influx and activation, T cell activation and cytokine production, B cell activation and antibody production			↓	↓*	↑	↓*	↓			↓		↓*	↓	↓*	
IL7R	The subunit of receptor for interleukin 7, which is essential for lymphocyte development and survival	↓*		↓				↓	↓			↓	↑*	↓*	↓*	
LST1	Membrane protein that can inhibit the proliferation of lymphocytes	↓*		↓				↓		↓			↓*			
LTB	Cytokine that forms complex with lymphotoxin-alpha and activates lymphotoxin-beta receptor. Associated with inflammatory response and apoptosis			↓*	↓*				↓	↓	↓	↓*	↓*	↓		
MALT1	A caspase-like protease that triggers NF-kappa B signaling and lymphocyte activation following antigen-receptor stimulation	↓*		↓	↓*	↓*						↓				
S1PR1	Receptor that modulates immune cells migration			↓*				↑			↓	↓	↑*	↓*	↓*	
TESPA1	Potentially participates in the positive regulation of T cell receptor signaling pathway	↓*		↓	↓*			↓	↓					↓*	↓*	
TNFRSF25	Receptor that stimulates NF-kappa B activity and regulates cell apoptosis, plays a role in regulating lymphocyte homeostasis			↓					↓	↓	↓	↓*		↓*	↓*	
TREM1	Receptor, which amplifies neutrophil and monocyte-mediated inflammatory responses by stimulating release of pro-inflammatory chemokines and cytokines, as well as increased surface expression of cell activation markers	↓*		↓*	↓*		↓*			↓		↑	↓*			↓
TSC22D3	Protects T cells from IL2 deprivation-induced apoptosis. In macrophages, plays a role in the anti-inflammatory and immunosuppressive effects of glucocorticoids and IL10	↓*		↓*		↑*							↓*	↓*	↓*	

^1^Immune function is a brief description of the gene function in immune cells derived from Uniprot database (https://www.uniprot.org). Asterisk (*) means that the gene is associated with adjusted p-value less than 0.05. Symbols ↑ and ↓ mean that the gene is up- or down-regulated, correspondingly.

Overall, **Figures 1**-**3** and **Table 2** demonstrate that PBMC associated with most of viral infection-related groups are characterized by increased proliferation, apoptosis, and altered immune functions (see the Discussion section for details).

### 3.3 Identification for receptors - master regulators that are common for different viral infections

**Figure 4:**
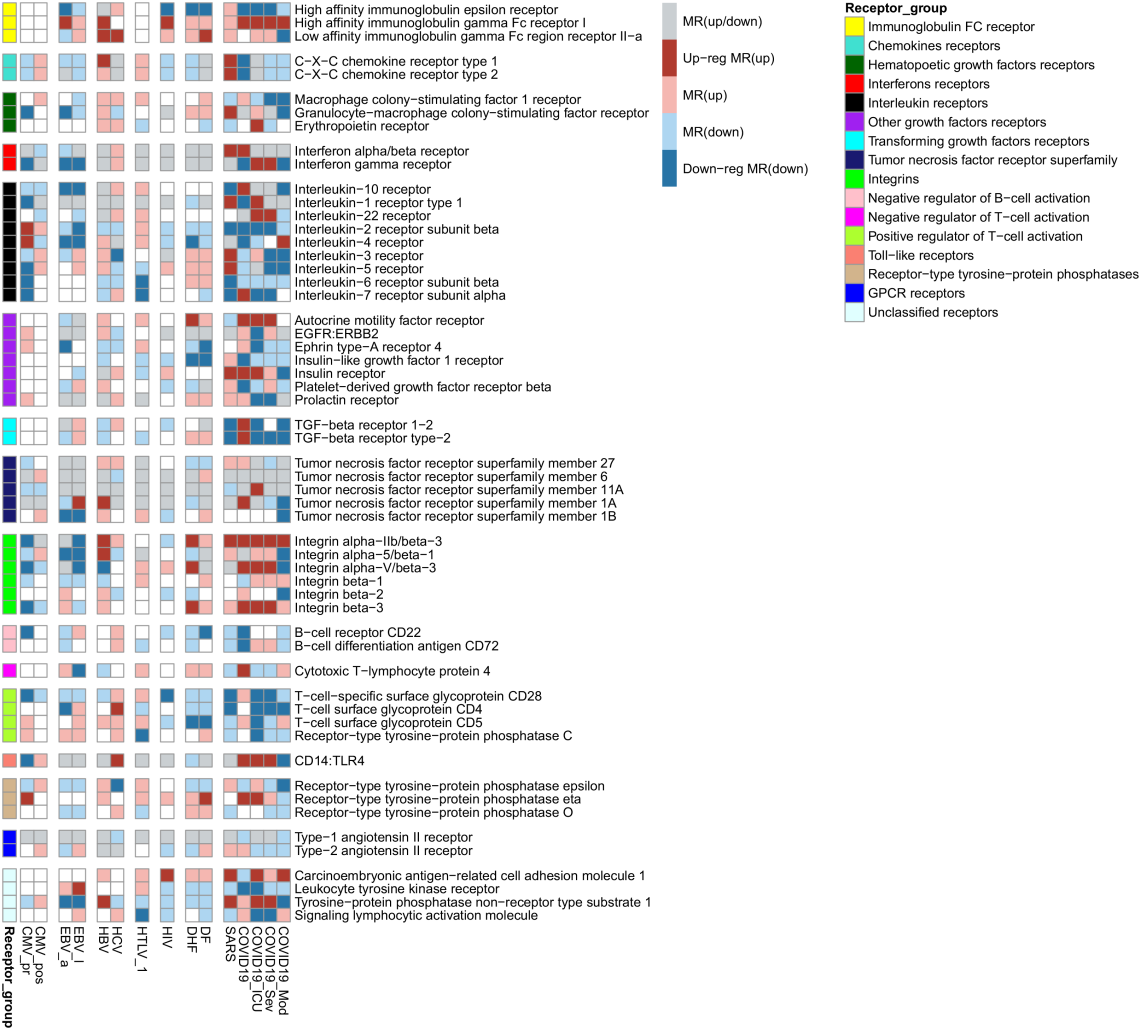
Receptors were identified as MRs for observed transcription changes in PBMC for at least six viral infections. The red color of cells means that the MR is responsible for the observed up-regulation of genes, dark red color means that the MR is responsible for the observed up-regulation of genes and is up-regulated itself, blue color means that the MR controls the observed down-regulation of genes, dark blue color means that the MR is responsible for the observed down-regulation of genes and down-regulated itself, grey color means that the MR controls both up- and down-regulation of genes.

We identified MRs using the Genome Enhancer pipeline (see Materials and Methods). MRs are the proteins at the top of the signalling network regulating the activity of transcription factors and their complexes and, in turn, are responsible for induction or maintenance of expression changes observed in PBMC. Most obtained MRs are intracellular “hubs,” such as kinases, phosphatases, ubiquitin ligases, GTPases, and transcription factors. We focused on receptors because their interaction with the corresponding ligands is the first of the consequent events leading to gene transcription changes. We selected 56 receptors identified as MRs for at least six viral infections. The receptors were grouped according to their belonging to families and functional similarities (**Figure 4**). **Figure 4** also demonstrates that MRs are up- or down-regulated themselves in part of viral infection-related groups. The full lists of transcription factors and MRs obtained by Genome Enhancer pipeline are presented in **Tables S5**, **S6**.

## 4 Discussion

To identify common molecular mechanisms associated with alteration of immune functions caused by both similar and different viral infections, we compared the transcription profiles in PBMC between pathologies caused by nine viruses: SARS-CoV-1, SARS-CoV-2, DENV, HIV-1, HTLV-1, HBV, HCV, CMV, and EBV. We estimated corresponding mechanisms at the levels of (i) smaller groups of functionally related genes (**Figures 1**, **3**), (ii) individual immune-related genes (**Table 3**), (iii) biological pathways (**Figure 2**), and (iv) receptors, the MRs that are the proteins at the top of signalling pathways responsible for the observed transcription changes (**Figure 4**).

The identified DEGs for each viral infection-related group reflect disease severity (DF or DHF, severe or moderate COVID-19) or duration (acute or latent EBV and CMV infections). There were hundreds DEGs for all groups except latent CMV infection that may indicate significant changes in functional states of immune cells in the corresponding pathologies. Interferon response has been shown to be important for immune-mediated defense process in viral infections that correspond to the earlier findings (47). Therefore, we checked the presence of ISGs among DEGs (**Figure 1** and **Table 2**). We found that most of viral infection-related groups of genes were associated with up-regulation of ISGs. The acute infections such as DF, EBV acute infection, untreated HIV infection and COVID-19, are associated with more up-regulated ISGs comparing to EBV, CMV and HTLV-1 latent infections. On the other hand, PBMC from patients with SARS and COVID-19 treated in Intensive Care Unit (ICU) are not associated with ISGs up-regulation (**Figure 1**). It was previously shown that type I interferon deficiency is associated with more severe COVID-19 (67). Such observations may indicate that the role of ISGs is significant in acute immune response but the interferon-induced immune response may decrease in time that leads to the development of severe pathology that requires ICU.

Pathway enrichment analysis allowed us to identify KEGG pathways associated with each viral infection-related group (**Figure 2**). Through the manual analysis of DEGs positions in the corresponding pathway maps, we identified the small groups of functionally related genes responsible for the regulation of the cell cycle, apoptosis, and immune cell activation and exhaustion (**Figure 3**). **Figures 2**, **3** demonstrate that all viral infections are associated with changes in these processes. The results obtained are in agreement with the known data on the mechanisms of the immune response to the studied viral infections. Antiviral response includes both non-specific innate immune response and adaptive immune response components (68). The innate immune response is typically mediated by the interferon activity (68), Toll-like receptors (TLRs), RIG-1 receptors (RLRs) signalling pathways and TANK-binding kinase 1 (TBK1) pathway (69). The TLR and TBK-1 pathways are involved in the molecular mechanisms of a viral infection during the early phase. Higher expression levels of interferons 1, 3, and 7 are shown in later stages and are responsible for the inflammation caused by a viral infection (68). The role of TLRs was also shown in activation of pro-apoptotic pathways in infected cells (70). Along with innate immune response, some specific molecular mechanisms are associated with each viral infection. In our study, we suggested that the changes in the transcription levels may reflect the pathways and molecular mechanisms involved in each specific viral infection and compared our results with earlier observations.

Primary CMV infection is known to cause lymphocyte activation and apoptosis, lymphocytosis, and immune exhaustion (25, 29, 65) and therefore it is associated with transcriptional changes that reflect the functional states of immune cells. It was demonstrated that in addition to the activation of pro-apoptotic mechanisms mediated by TLRs, CMV-infection also leads to the changed expression of the cellular anti-apoptotic protein Mcl-1, a member of bcl-2 family (71). This process, in turn, results in an apoptosis of monocytes. HIV infection is associated with the up-regulation of cyclins A, B, and E. Since PBMC are non-synchronized in cell cycle phases, this observation may indicate cell cycle arrest in the G2 phase (72, 73). Down-regulation of the co-stimulation receptor CD28 and increased transcription of the immune checkpoint CTLA4 indicates that HIV infection causes activation and exhaustion of T-lymphocytes. The “exhausted” state of T-cells is characterized by increased expression of immune checkpoints, disrupted proliferation, decreased functions, and increased apoptosis (28, 72). Chronic HCV infection is associated with the disruption of the NK cells functions and the exhaustion of immune cells (74, 75). The number of DEGs related to above mentioned processes increase with disease severity. The DHF is associated with more DEGs related to apoptosis and decreased NK cell functions than DF. It was demonstrated that HTLV-1 can interact with components of NF-kappa B pathway, leading to deregulation of apoptosis (76). Another signalling pathway affected by HTLV-1 is AP-1 signalling pathway, whose components control cell activation and proliferation (76). SARS and severe COVID-19 associated with more transcription changes related to T, B and NK cells functions alterations than moderate coronavirus infection. The T cells are dysregulated through CD147 and receptor tyrosine kinase AXL (77). In particular, the expression of these two molecules was shown to be increased in CD4+/CD8+ T-cells in peripheral blood (77).

Further we provide some examples of genes (**Table 3**), which are up- or down-regulated by at least five viral infections, and involved in the host immune response to several different viral infections. The relationships of these genes to viral infections are less studied, and they represent new findings. The most of them are down-regulated by viral infections, but some of them are up-regulated. In particular, up-regulated lymphocyte antigen 6 family member E (LY6E) is a surface protein that regulates host immune response including enhancement of T-lymphocytes activation and proliferation (78). LY6E inhibits entry of SARS-CoV, SARS-CoV-2 and MERS-CoV into cells by interfering with spike protein-mediated membrane fusion (79), but promotes entry of HIV-1, and DENV. C-X-C motif chemokine ligand 10 (CXCL10) is involved in proinflammatory signal pathways, including chemotaxis, differentiation, and activation of immune cells, and immune response to viral infections (80, 81). The corresponding gene is up-regulated in five viral infections (**Table 3**). It is known that the increased level of CXCL10 is associated with COVID-19 severity (82). Earlier studies also confirm the involvement in immune response for several molecules that correspond to DEGs revealed in our study, including SEC14 like lipid binding 1 (SEC14L1). C-C motif chemokine receptor 7 (CCR7), and CD83 (83–85). CD96 downregulates inflammatory and cytokine response causing by NK cells (86). It is interesting, the expression of CD96 is decreased in some samples obtained from patients infected with DENV, coronaviruses SARS and SARS-CoV-2 and HBV. Therefore, one may suggest that for these infections cytokine inflammatory response can be caused by inhibition of CD96 function among some other signal pathways. The proteolytic activity of MALT1 paracaspase is required for T cell activation (87). It also plays role in reactivation of HIV-1 (88). It was demonstrated that MATLT1 inactivates NEDD4-binding protein 1 (N4BP1) that inhibits HIV-1 development in primary T cells and macrophages (88).

Therefore, the identified molecules can be considered as the biomarkers for the severity of viral disease progression. Also targeting molecules by direct interaction or modulation by gene expression may be considered as one of novel strategies for search for non-specific antiviral agents.

Since the observed transcriptional changes are associated with well-known immunopathogenesis of studied viral infections, we searched for MRs, which are the proteins at the top of signaling networks and are responsible for the maintenance of observed transcriptional changes. Our study focused on receptors because their interaction with corresponding ligands is the first of the consequent events leading to gene transcription changes. We identified many cytokines and growth factors receptors, which are common for studied viral infections (**Figure 4**). According to Human Proteins Atlas data^7^, all of them are expressed in PBMC: lymphocytes, monocytes, or NK cells. The revealed receptors are up- or down-regulated themselves in part of viral infection-related groups (**Figure 4**). It means that they are part of positive feedback loops and are extremely important to maintain the observed transcription profiles.

The revealed receptors differ in the novelty of their relationships with the functional states of studied immune cells, they can be divided into three groups by this criterion. The receptors of the first group that include interferons alpha and gamma, and interleukins 1-10, have a clear relationship with functional states of PBMC. They are essential for cell activation, differentiation, survival, and proliferation. The role in immune response of the receptors belonging to the second group is under active investigation. Such receptors include immunoglobulin gamma and epsilon receptors and C-X-C chemokine receptors type 1 and 2 (receptors for interleukin 8). For example, CXCR1 and CXCR2 genes encode receptors for interleukin 8, which is well known as a potent neutrophil chemotactic factor. The CXCR1 receptor is also expressed on the cell surface of T cells, and interleukin 8 treatment results in a reduction in the activation status of both effector memory T cells and terminally differentiated effector T cells (89). Interleukin 8 is also an essential chemotactic factor for NK cells (90) and reduced expression of CXCR1 is associated with decreased counts of NK cells in lymph nodes and incomplete control of HIV viral replication during the early stages of the disease (91). The FCGR2A gene encodes low-affinity immunoglobulin gamma Fc region receptor II-a that binds to the Fc region of immunoglobulins gamma and promotes phagocytosis of opsonized viral particles (92, 93). Polymorphisms in the FCGR2A gene are associated with severity and outcome of viral infections such as COVID-19 (94), Influenza A (95), and HIV infection (96, 97). Surprisingly, we also identified high-affinity immunoglobulin epsilon receptor, which is well known to be expressed in mast cells and basophils and is responsible for initiating the allergic response. However, it was recently shown that CD4(+) T cells also express immunoglobulin epsilon receptors that induces production of interleukin 6 and interferon-gamma but reduces production of interleukin 10 (98). Polymorphism in the FCER1A gene, which encodes the alpha subunit of the receptor, is associated with the efficacy of HBV infection treatment by peginterferon alfa-2a (99).

The third group of the revealed receptors are not specific to the immune system, and their associations with immune response are not studied enough. For instance, the role of Ephrin type-A receptor 4 is well described for the nervous system; however, it is also expressed on memory T cells and is responsible for their migration (100). The insulin receptor is essential for normal adaptive immune function through modulating T cell nutrient uptake and associated glycolytic and respiratory capacities (101). Moreover, following insulin binding, the insulin receptor translocates to the nucleus, where it plays a crucial role in regulating the transcription of various immune-related genes, including pathways involved in viral infections. Therefore, diabetes with insulin resistance can directly contribute to an inadequate immune response, e.g., observed during COVID-19 (102) and some other severe acute viral infections. Prolactin and its receptors play an important role in regulating innate and adaptive immune responses (103), particularly in T lymphocyte growth and activation (104, 105). Stimulation of angiotensin AT1 receptors is important for T cell activation and adhesion/transmigration through the basal endothelial membrane, whereas angiotensin AT2 receptors limit this effect (106, 107). AMFR gene encodes the receptor for autocrine motility factor, the protein with multiple functions. It is little known about its functions in the immune cell. Particularly, the autocrine motility factor stimulates immunoglobulin secretion by cultured human PBMC (108).

We revealed new untrivial associations between the human genes and alterations in immune response, which are common for several viral infections. Nevertheless, this study has some limitations. First, the transcription datasets used in the study (**Table 1**) are highly heterogenic. They may contain unknown or hidden confounders that may influence the results. Such demographic characteristics as age, gender and geographic region cannot be taken into account in our study because the corresponding information is not available for the most of used datasets. Some datasets were coming from RNAseq studies, some others - from microarrays of different platforms allowing measuring transcription of different numbers of genes. This issue might be solved using normalization procedure, however different number of target genes in microarray and RNAseq studies comprises the limitation of the study. The heterogeneity of datasets may have introduced some biases in the obtained results. Second, the results of the analysis represent rather statistical than causal relationships. The causal nature of revealed relationships can be analysed using additional experiments in cell cultures or animals. Third, the results were obtained at the level of transcripts, however the analysis of the host response to viral infections at the protein level is currently restricted due to the lack of proteome experimental data. Nevertheless, identified DEGs and MRs can be considered as the key features common for several viral infections and can be used as a basis for the development of novel therapeutic strategies for pathogenesis-directed antiviral therapy. New therapeutic strategies developed using the results of computational analysis may include the use of drugs with pathogenesis-based mechanisms of action, including those that can be found by drug repurposing. In this case, identification of human genes and corresponding proteins, as well as the molecular mechanisms controlled by them, may be useful in the search for new potential therapeutic strategies. Furthermore, identifying common mechanisms among various viral infections may help to understand the possible role of viral infections in the development of other diseases, such as cancer.

## 5 Conclusions

Since many different viruses currently exist and new viral infections, such as COVID-19, suddenly become spreading among human population, it is challenging to create direct therapeutics for each virus. Additionally, complications can develop rapidly after a person contacts a virus. In such cases pathogenesis-directed therapy may be beneficial for treatment and cure of severe viral infections. Even though significant differences between viral diseases exist, most of them are associated with the dysfunction of the immune system. As a result, the immune system cannot effectively eliminate viruses, which increase disease severity and duration and susceptibility to secondary infections. Thus, it is crucial to identify mechanisms of immune system disruption that are common for different viral infections. In our study, we applied network-based transcriptomics analysis to identify mechanisms of immune function disruption caused by nine different viral infections at the levels of pathways, cellular processes, and master regulators, which are the key proteins responsible for the observed immune states. Analysis of revealed pathways, small groups of functionally related genes and individual differentially expressed genes demonstrated that nine studied viral infections cause immune activation, exhaustion, cell proliferation, and increased susceptibility to apoptosis for peripheral blood mononuclear cells. Additionally, we performed a search for receptors – master regulators, which are at the top of immune cells signalling networks and may be responsible for the maintenance of observed transcriptional changes, and, thus, accountable for the observed changes in immune cell state. Besides well-characterized receptors for interleukins and interferons, we identified receptors whose relationships with virus-induced immune disfunction have not been shown earlier. The receptors for autocrine motility factor, insulin, prolactin, angiotensin II, and immunoglobulin epsilon may be essential for the normal functioning of immune cells during anti-viral response. They regulate immune cell activation, growth, metabolism, migration and secretion of immunoglobulins and cytokines, and may be involved in the maintenance of gene transcription changes, induced by different viral infections and associated with altered immune response. The pharmacological modulation of the receptors may change transcription profiles in immune cells, which may lead to recovery of immune functions and increase the efficacy of anti-viral response. Thus, the revealed receptors may be investigated as potential targets for treating severe viral infections.

## Data availability statement

The original contributions presented in the study are included in the article/**Supplementary Material**. Further inquiries can be directed to the corresponding author.

## Ethics statement

As publicly available data was used in this analysis, ethical approval was not required.

## Author contributions

SI, OT, and VP contributed to conception and design of the study, and reviewed and prepared the final version of the manuscript. SI and OT prepared transcription datasets. SI wrote R scripts and performed analysis with Genome Enhancer pipeline. SI and OT wrote the first draft of the manuscript. SI, OT and VP reviewed and prepared the final version of the manuscript. All authors contributed to the article and approved the submitted version.

## Footnotes

1
https://www.ncbi.nlm.nih.gov/geo

2
https://www.ebi.ac.uk/arrayexpress

3
https://ge.genexplain.com

4
https://www.genome.jp/kegg/pathway.html

5
https://CRAN.R-project.org/package=pheatmap

6
https://www.uniprot.org

7
https://www.proteinatlas.org

## Supplementary material

Supplementary Table 1 Differentially expressed genes found for particular viral infection-related groups.

Supplementary Table 2 Summary on differentially expressed genes.

Supplementary Table 3 The list of 50 interferon-stimulated genes.

Supplementary Table 4 The full lists of KEGG pathways found for particular viral infection-related groups.

Supplementary Table 5 Transcription factors are potentially responsible for observed gene expression changes in particular viral infection-related groups.

Supplementary Table 6 Master regulators are potentially responsible for observed gene expression changes in particular viral infection-related groups.
